# Comparison of TLR4 Genotype and TLR4 Pathway-Related Cytokines in Different Strains of Mice in Response to Pertussis Toxin Challenge

**DOI:** 10.3390/genes15111435

**Published:** 2024-11-05

**Authors:** Jie Wei, Lichan Wang, Chen Wei, Jiaona Guang, Hong Wang, Jiaqi Zhou, Huan Li, Xiao Ma, Bingfei Yue

**Affiliations:** 1 Division of Laboratory Animal Monitoring, National Institutes for Food and Drug Control, Beijing 102629, China; weijie@nifdc.org.cn (J.W.); guangjiaona@joinn-lab.com (J.G.); wanghong@nifdc.org.cn (H.W.); jhan90657@gmail.com (J.Z.); huanl24414@gmail.com (H.L.); 2 China National Rodent Laboratory Animal Resources Center, Beijing 102629, China; 3 Division of Diphtheria, Tetanus and Pertussis Vaccine and Toxins, National Institutes for Food and Drug Control, Beijing 102629, China; 4 Key Laboratory of the Ministry of Health for Research on Quality and Standardization of Biotech Products, Beijing 102619, China

**Keywords:** toll-like receptor 4, pertussis toxin, cytokines, genotype

## Abstract

Background: The genetic background of Toll-like receptor 4 (TLR4) proved to be important in the induction of immune protection against *Bordetella pertussis* infection in humans. Currently, the evaluation of the acellular pertussis (aP) vaccine depends largely on using different mouse strains, while the TLR4 genotype of different mouse strains in response to pertussis toxin (PT) is not carefully determined. The current study was designed to determine the differences in TLR4 genotype and TLR4 pathway-related cytokines in response to PT stimulation among mouse strains of ICR, NIH, and BALB/c. Method: We first determined the single-nucleotide polymorphisms (SNPs) in the TLR4 gene by using first-generation sequencing. Then, the cellular response, including the TLR4 mRNA expression and TLR4 signaling-related cytokines, of immune cells from different mouse strains after PT stimulation was determined. Result: Three missense mutation sites (rs13489092, rs13489093, rs13489097) of the TLR4 gene were found. ICR mice were homozygous without mutation, NIH mice were partially heterozygous, and BALB/c mice were homozygous with a missense mutation. The expression of TLR4 was repressed while the downstream cytokines were upregulated after PT stimulation differently among mouse strains. The IFN-β cytokine of the TRIF pathway was significantly increased in ICR mice (*p* < 0.05). The IL-6 cytokine of the MyD88-dependent pathway was significantly increased in BALB/c mice (*p* < 0.05). The identified SNPs of the TLR4 gene in different mouse strains might account for the differences in cytokines levels determined after PT stimulation. Conclusions: Our studies might provide useful referees to reduce the mouse-derived difference in the determination of vaccine titer and increase the comparability of the vaccine from different origins, as different mouse strains were used for vaccine development in different countries.

## 1. Introduction

Whooping cough, also known as pertussis, is induced by the infection of the Gram-negative bacterium *Bordetella pertussis (B. pertussis)*. Currently, the occurrence and progression of pertussis can only be partly limited by whole-cell or acellular pertussis (aP) vaccines. Also, the widespread population vaccination to *B.p* cannot prevent the endemic and re-emergence of such an infection, either. Pertussis toxin (PT) is the only internationally recognized protective antigen in acellular pertussis vaccines [1,2,3], with the protective efficacy of other components such as filamentous hemagglutinin (FHA) and lipopolysaccharide (LPS) still being debated. As aP vaccines are currently the mainstream in pertussis immunization, studying the mechanism of this primary effective component, PT, is of paramount importance. Therefore, further exploration of the mechanism underlying the infection sensitivity to *B.p* is still urgent. Previously, researchers have found that the variation in the gene coding for Toll-like receptor 4 (TLR4) is closely related to the infection sensitivity to *B.p* and vaccine response to the whole-cell pertussis vaccine. However, the genetic background of TLR4 in response to acellular pertussis vaccine challenge has not been carefully evaluated in mice used for vaccine development.

The immune status of mice to the vaccine is determined by the genetic background and environmental factors, in which the genetic background of the organism plays a decisive role. Using genome-wide analysis of inbred mice, researchers found multiple genes linked to the lifespan of anti-pertussis antibodies produced by the body [4]. Of all the genes that have been reported to affect the immune response to the pertussis vaccine, the TLR4 gene has been mostly studied in humans [5,6,7]. The combination of PT and TLR4 has been considered to be the main pathway for PT to stimulate immunity, such as experimental encephalomyelitis caused by PT. However, studies to determine the association between TLR4 gene locus and pertussis vaccine immune response are limited to clinical analysis. Studies have shown that the antibody titer of the TLR4 polymorphic population is significantly higher than that of the TLR4 monomorphic population. The single-nucleotide polymorphisms (SNPs) loci of human TLR4 (rs2770150, rs6478317, rs4986790) are significantly associated with the level of specific antibodies against pertussis toxin [8,9,10]. TLR4 is a conserved gene with high similarity between humans and mice. Therefore, by utilizing the commonly applied mouse strains in aP vaccine evaluation, the current study firstly determined the genetic variations in TLR4 in different mouse strains; subsequently, by comparing the expression of TLR4 and its related cytokines in response to PT, our study might provide useful referees to reduce the mouse-derived difference in the determination of vaccine titer and increase the comparability between the titer and the true protection of the vaccine.

## 2. Materials and Methods

### 2.1. Animals

This experiment has been approved by the Animal Welfare Ethics Committee of the National Institute for Food and Drug Control of China (2020-B-012). The mice used were 5-week-old and half-sex. According to the third part of the Chinese Pharmacopeia (2020 edition), the modified intracerebral challenge assay (MICA) is used for aP vaccine potency evaluation, while NIH mice are the recommended strains. However, ICR mice and pertussis serological potency test (PSPT) methods were recommended by the European Pharmacopeia [11]. The US federal regulations for aP vaccine evaluation only provide guidelines without detailed operating procedures. Meanwhile, as the established intranasal infection model, BALB/c mice have been widely used as a research model for evaluating the potency of new aP vaccines [12,13]. Therefore, in this study, 20 ICR mice were purchased from Vital River Laboratory Animal Technology Co., Ltd., Beijing, China, and 20 NIH and 30 BALB/c mice were obtained from the laboratory animals center of the National Institute for Food and Drug Control of China. Among them, 10 BALB/c mice were used in the pretest of stimulation time and concentration with aP vaccine or PT antigen. Animal vaccine immunization was performed in barrier facilities with negative pressure at the National Institute for Food and Drug Control of China.

### 2.2. Vaccine and Immunization

The vaccines tested in this study were Diphtheria, tetanus, pertussis (acellular, component), poliomyelitis (inactivated) vaccine, and Haemophilus type B conjugate vaccine, adsorbed (DTaP/IPV), containing pertussis toxin (PT) and filamentous haemagglutinin antigens (FHA), 50 μg/mL, respectively. All tested vaccines were provided by the National Institute for Food and Drug Control (NIFDC) of China, have undergone endotoxin and pyrogen testing, are qualified for release, and are free from LPS contamination.

A total of 10 mice in each strain were injected with sterile saline and defined as the control group. The dose of intraperitoneal injection was 0.5 mL/mouse. And the other 10 mice were intraperitoneally injected with 0.5 mL of aP vaccine 5-fold diluted, defined as the immunized group.

### 2.3. SNP Selection and Genotyping

The potential mutated SNP sites on the exons of the TLR4 gene were searched on the UCSC website (http://genome.ucsc.edu/, accessed on 24 July 2023), and the location of the SNP sites in the gene was recorded. Gene fragments not longer than 800 bp and covering as many SNP loci as were obtained on NCBI were obtained and subjected to Primer 5.0 software for primer design. The selected SNPs and corresponding detecting primers are listed in Table 1.

The genomic DNA was exacted from mice tails according to the manufacturer’s instructions (Qiagen). DNA was amplified in a 20 μL volume using 13.8 μL ddH_2_O, l μL of 2.5 mmol/L dNTP, l μL of each primer, 0.2 μL of 5 U/μL Taq enzyme, 2 μL of 10 × Mg^2+^ buffer, and 50 ng of DNA. The amplification process was performed on Applied Biosystems (ABI) as follows: 94 °C 5 min; 30 cycles of 94 °C 30 s, 30 s at optimized annealing temperature, and 30 s at 72 °C; and finally 7 min at 72 °C. The harvested fragments were then subjected to first-generation sequencing performed by Sangon Biotech (Shanghai, China) Co., Ltd. The SNPs were identified by comparing the harvested gene sequence to the reference genome from NCBI.

### 2.4. Choice of Stimulation Time with PT Antigen

The spleens of 5 BALB/c mice were harvested under sterile conditions. The spleens were cut into small pieces using sterile surgical scissors and then pressed through a 70 µm cell strainer (BD Biosciences, East Rutherford, NJ, USA) using a sterile syringe plunger to obtain a single-cell suspension. The cells were then centrifuged at 300× *g* for 5 min at room temperature. The supernatant was discarded, and the cell pellet was resuspended in serum-free medium for lymphocytes (SuperCulture™ L500, Dakewe Biotech Co., Ltd., Shenzhen, China).

The cell density was adjusted to 5 × 10^6^ cells/mL. For stimulation, the lymphocytes were treated with detoxified PT antigen of 5 µg/mL in a 24-well plate. The cells were incubated at 37 °C in a 5% CO_2_ atmosphere for 24, 48, and 72 h.

After the stimulation period, the cells were collected for subsequent analysis, including RNA extraction and flow cytometry, to assess lymphocyte activation markers. The PT protein used in this study was detoxified and retained properties similar to the PT component found in commercially available vaccines.

### 2.5. Choice of Stimulation Concentration with PT Antigen or aP Vaccine

Multiple concentration gradients of PT antigen were prepared in serum-free medium: 0 μg/mL, 1 μg/mL, 2.5 μg/mL, and 5 μg/mL. Concurrently, aP was diluted 5-fold, 10-fold, and 25-fold to match the PT antigen content in the vaccine with that of the standalone PT antigen.

Under sterile conditions, splenic lymphocytes were extracted from five BALB/c mice and the splenic lymphocytes (approximately 5 × 10^6^ cells) were stimulated with the different concentrations of PT antigen and aP for 48 h, with microscopic observations at 24 h and 48 h.

After 48 h of stimulation, the culture wells were gently tapped to recover the lymphocytes from both the PT group and the vaccine group (the recovery rate should be above 90%). This was followed by rinsing with 1 mL PBS 1–2 times, ensuring that the rinsed cell suspension was completely transferred to a 15 mL centrifuge tube, and centrifuging at 300× *g* for 5 min. The supernatant was discarded, and RNA was immediately extracted from the underlying lymphocytes.

Subsequently, the RNA was reverse transcribed into cDNA and quantitative PCR (Q-PCR) was used to detect the relative expression of the TLR4 gene. The concentration of the stimulant where the relative expression of the TLR4 gene changes significantly before and after stimulation was selected.

### 2.6. RT-PCR

After the stimulation period, the total RNA from the lymphocytes was extracted using the Trizol reagent (Thermo Fisher Scientific, Waltham, MA, USA) according to the manufacturer’s instructions. Briefly, the cell pellet was resuspended in 1 mL of Trizol, and the cells were lysed by pipetting up and down. Following lysis, 200 µL of chloroform was added, and the mixture was shaken vigorously for 15 s before allowing it to settle for 2–3 min at room temperature. The aqueous phase was carefully collected, and RNA was precipitated by adding 500 µL of isopropanol. The RNA pellet was washed with 75% ethanol, air-dried, and resuspended in RNase-free water.

A total of 1 μg of total RNA was then subjected to reverse transcription using the Quanti Tect Reverse Transcription Kit (Qiagen, Dusseldorf, Genmany) according to the manufacturer’s protocol. The cDNA was diluted to a final volume of 60 µL for quantitative RT-PCR.

Quantitative RT-PCR was performed using SYBR Green Master Mix (Thermo Fisher Scientific, Waltham, MA, USA) on an Applied Biosystems 7500 Fast Real-Time PCR System. The amplification was carried out using specific primers for TLR4 (Assay ID: Mm00445273_m1) and GAPDH (Assay ID: Mm03302249_g1) as an internal control. The PCR conditions were set to preheat at 50 °C for 2 min, initial denaturation at 95 °C for 10 min, followed by 40 cycles of denaturation at 95 °C for 15 s, and annealing at 60 °C for 1 min. The relative expression of TLR4 was determined using the 2^−ΔΔCt^ method and compared with the GAPDH control group.

### 2.7. Cytokines Determination

The 1640 medium (Gibco, Cat. No. C22400500BT) with 5% fetal bovine serum was used in specific stimulation with PT (100 μL of the stimulator and an equal volume concentration of 10^6^ cells/mL of spleen lymphocyte suspension were added to a 96-well plate and then stimulated for 12 h at 37 °C for the assay). After the PT stimulation, cells were centrifuged, and the supernatants were collected for determining the expression of TLR4 signaling-related cytokines. The lymphocyte grouping and the levels of cytokines secreted were detected by flow cytometry. Biolegend kit (Cat. No. 740150) was used for IL-12 p70, IL-1β, IL-6, and IFN-β expression.

### 2.8. Statistical Analysis

The results were presented as means ± standard deviations ($\overset{-}{x}$ ± s). Statistical analysis was performed using SPSS 21.0. Student’s t-test was used to compare the relative TLR4 expression and cytokine levels of different strains of mice before and after PT stimulation. A *p* < 0.05 was defined as statistically significant.

## 3. Results

### 3.1. TLR4 Genotyping in Different Strains of Mice

We have selected 10 SNP sites that may have missense mutations in the TLR4 gene of ICR, NIH, and BALB/c mice. As determined by genotyping and subsequent sequencing, the SNP loci of TLR4 of different strains of mice were different at rs13489092, rs13489093, and rs13489097 (Table 2). Among them, ICR mice were homozygous without mutation, NIH mice had partial heterozygous genotypes, and BALB/c mice had homozygous missense mutations.

### 3.2. Differences in TLR4 Gene Expression Before and After PT Stimulation

**Figure 1:**
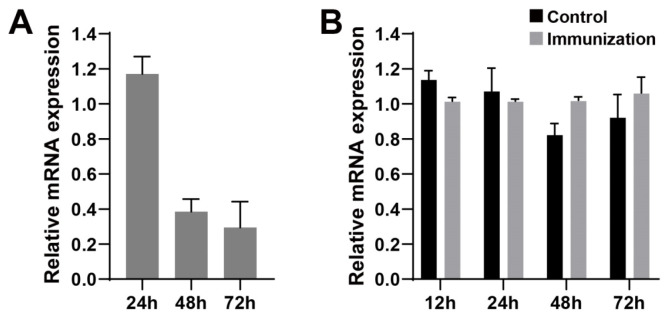
Changes in TLR4 gene expression in mouse spleen lymphocytes after PT or aP vaccine stimulation. Note: (**A**): The changes in TLR4 gene expression in spleen lymphocytes of BALB/c mice after PT antigen stimulation for 24 h, 48 h, and 72 h. Sample size: *n* = 5. (**B**): Changes in TLR4 gene expression in splenic lymphocytes of BALB/c mice 12 h, 24 h, 48 h, and 72 h after intraperitoneal 5-fold diluted aP vaccine immunization. Sample size: *n* = 5.

The expression of the TLR4 gene in spleen lymphocytes of BALB/c mice stimulated by PT antigen (5 μg/mL) for 24 h, 48 h, and 72 h was compared. It was found that PT antigen had significantly inhibited the expression of the TLR4 gene at 48 h. At the same time, BALB/c mice were immunized with the 5-fold diluted vaccine in vivo for 12 h, 24 h, 48 h, and 72 h. The changes in TLR4 gene expression levels in spleen lymphocytes of the vaccine group and the control group were detected. The results showed that there was no significant difference in TLR4 gene expression levels before and after immunization (*p* > 0.05, Figure 1).

**Figure 2:**
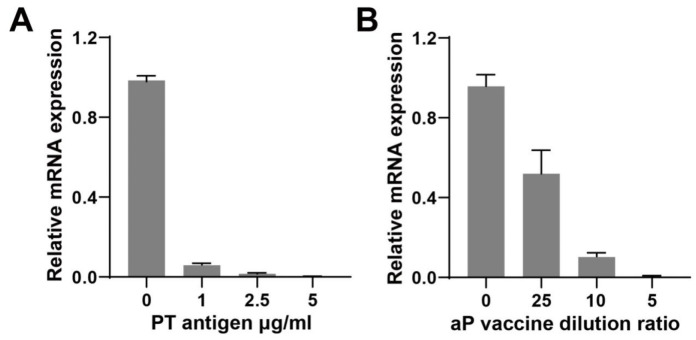
Expression level of the TLR4 gene under different concentrations of PT antigen or aP vaccine stimulation. Note: (**A**): The changes in TLR4 gene expression in splenic lymphocytes of BALB/c mice after being stimulated with different concentrations of PT antigen. (**B**): Changes in TLR4 gene expression in spleen lymphocytes of BALB/c mice after stimulation with different concentrations of aP vaccine. Sample size: *n* = 5.

The spleen lymphocytes of BALB/c mice were stimulated with different concentrations of PT antigen and multiple dilutions of aP for 48 h, and the changes in TLR4 gene expression level were compared. The results showed that the concentration of the stimulator was negatively correlated with the expression level of the TLR4 gene in both PT antigen and aP, as shown in Figure 2. When the concentration of PT antigen was 5 μg/mL, the inhibition of TLR4 gene expression was the highest (Figure 2).

The expression of TLR4 was also determined in PT antigen (5 μg/mL)-stimulated lymphocytes from ICR, NIH, and BALB/c mice after 48 h. The results showed that the expression levels of the TLR4 gene in three strains of mice were significantly decreased after PT stimulation (*p* < 0.05). The degree of TLR4 inhibition in different strains of mice was compared, and it was found that BALB/c mice had a higher degree of inhibition, as shown in Table 3. Analysis of variance showed that there were significant differences between ICR mice, NIH mice, and BALB/c mice (*p* < 0.05), but there was no significant difference among the untreated group of the mouse strains determined (*p* > 0.05).

### 3.3. Changes in TLR4 Pathway-Related Cytokines Before and After PT Stimulation

**Figure 3:**
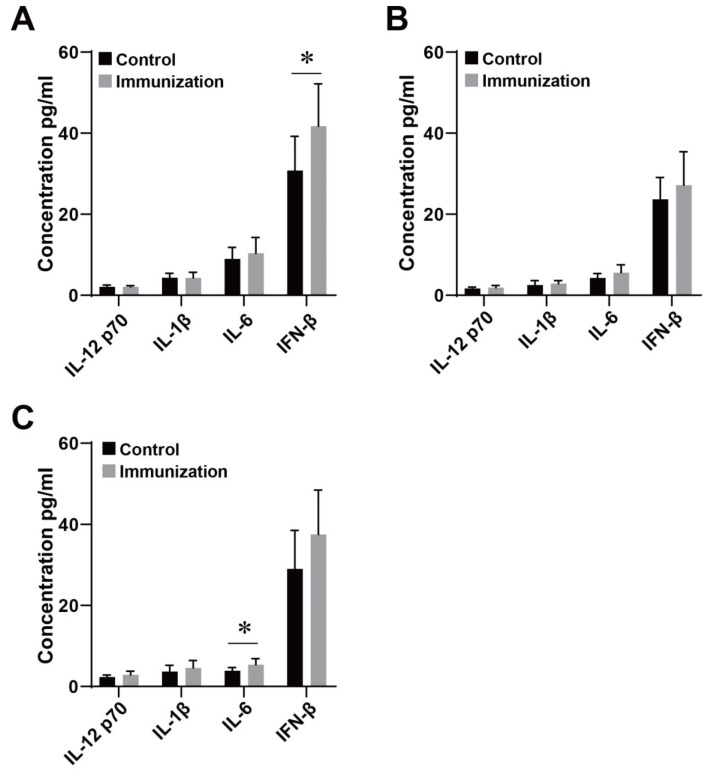
Changes in TLR4 pathway-related cytokines after PT stimulation. Note: The contents of TLR4 pathway-related cytokines in spleen lymphocytes of ICR (**A**), NIH (**B**), and BALB/c (**C**) mice after PT immune stimulation. “*” indicates *p* < 0.05. Sample size: *n* = 10.

After binding to TLR4, PT can activate related cytokines through the TLR4 protein-mediated MyD88-dependent pathway and the TRIF pathway. The concentration of cytokines related to the TLR4 pathway in spleen lymphocytes of ICR, NIH, and BALB/c mice after PT stimulation is shown in Figure 3. After PT stimulation, TLR4 pathway-related cytokines showed an up-regulating trend, and IFN-β in ICR mice increased significantly. The IL-6 of BALB/c increased significantly. There was no significant difference in cytokines of NIH mice before and after immunization.

## 4. Discussion

TLR4, one of the most intensively investigated pattern recognition molecules, is widely distributed on the cell membrane of lymphocytes and epithelial cells [14]. Studies have shown that the binding of the pertussis vaccine could activate the NF-κB signaling, promoting the production of a variety of cytokines, which not only can act on antibody generation but also can induce the activation of dendritic cells, thus affecting the overall adaptive immune response process [15]. Banus et al. [16] found three SNP sites on the TLR4 gene of inbred mice that may be related to the susceptibility of *B.p*, among which rs3023006 is a missense mutation site on the exon. The occurrence of a missense mutation in the exons of a gene could cause changes in amino acids, which may alter the structure of the protein and its function. Our results showed that ICR, NIH, and BALB/c mice were homozygous at rs3023006. Pellegrino et al. [17] summarized the SNP loci that may be related to the immune response of the pertussis vaccine in the human TLR4 gene. Sander Banus [10] proved that the rs2770150 locus of the human TLR4 gene may be related to the level of PT-IgG, but there is no research showing the relationship between TLR4 genotyping and pertussis vaccine immune response in mice. In this study, the sequencing results of the TLR4 gene in ICR, NIH, and BALB/c mice were compared and analyzed. Among them, the three strains of mice were significantly different in the three missense mutation sites of rs13489092, rs13489093, and rs13489097. However, whether these three SNP loci affect the specific antibody content of the pertussis vaccine needs further research support.

In addition to genotyping, gene expression levels may also affect gene function. Maria Nasso et al. [18] cultured HEK293 cells with PT antigen stimulation, detected the expression of the TLR4 gene before and after stimulation, and found that the expression level of the TLR4 gene increased with the increase in PT antigen concentration. The activation of the TLR4 pathway is the main pathway by which PT causes autoimmune diseases as an adjuvant [18,19,20]. In recent years, scientists have found that PT could evoke not only immune enhancement but also immunosuppressive responses [21]. Vadasz et al. [22] found that the expression level of the TLR4 gene in lymphocytes of children infected with pertussis was lower than that of uninfected pertussis infants. This phenomenon is also reflected in the study of the pertussis vaccine as reported by Carbonetti et al. [23], in which they found that the colonization rate of pertussis bacteria with PT antigen was higher than that of wild pertussis bacteria in the mouse nasal infection model. The change in TLR4 gene expression in lymphocytes under the action of PT antigen is still inconclusive. Therefore, in this study, PT antigen was used to stimulate mouse spleen lymphocytes, and aP was used to immunize mice in vivo and in vitro to detect the expression level of the TLR4 gene. The results showed that there was no significant difference in the expression level of TLR4 gene in spleen lymphocytes of BALB/c mice before and after immunization at 12 h–72 h. When lymphocytes were stimulated in vitro, both PT antigen and aP could significantly inhibit the expression of TLR4 gene, and the degree of inhibition increased with the increase in stimulator concentration. This may be a way for PT as a part of pertussis bacillus to exert immunosuppressive effects locally. We believe that in vivo methods reflect the overall immune response to the vaccine, while in vitro methods focus more on the impact of PT as a single factor on immunity, allowing for a clearer exploration of the mechanisms of individual components. This result is inconsistent with the conclusion of Maria Nasso’s [2] research, which may be caused by the different cells used in the experiment.

In this study, GAPDH was used as an internal reference to detect the Ct value of the TLR4 gene, and the expression level of the TLR4 gene was expressed by 2^−ΔΔCt^. The Ct value is the number of cycles required when the fluorescence intensity in the PCR tube reaches a threshold [24]. A total of 40 cycles were performed in the qRT-PCR part of this experiment. When the Ct value of TLR4 was higher than 35, the detection results may be biased [25]. Therefore, in this study, the concentration of the cDNA template was increased to make the Ct value of TLR4 within 35. Previous experiments showed that the expression of the TLR4 gene in mouse lymphocytes was inhibited after PT antigen stimulation. When the concentration of PT stimulation was 5 μg/mL and the stimulation time was 48 h, the inhibitory effect was the most significant. The changes in the TLR4 gene expression level in spleen lymphocytes of ICR, NIH, and BALB/c mice before and after PT antigen stimulation were compared and analyzed. Among them, BALB/c mice had the strongest inhibition.

Studies have shown that TLR4 can not only bind to lipopolysaccharides but also bind to PT and even have a higher affinity than LPS when the body is immunized with the pertussis vaccine [26]. Therefore, the binding of TLR4 to PT antigen is more important in the immune process of aP. After binding to TLR4, PT antigen can activate the NF-κB pathway through the MyD88-dependent pathway and promote the production of pro-inflammatory cytokines such as IL-12, IL-1, and IL-6. It can also promote the secretion of IFN-β through the TRIF pathway, and the activation process is similar to the LPS activation process [9,27,28]. After PT antigen stimulation, the levels of IL-6 and IFN-β produced by spleen lymphocytes of different strains of mice were different. The results showed that IFN-β in ICR mice increased significantly, and the level of IL-6 in BALB/c mice increased significantly. The levels of TLR4 pathway-related cytokines in NIH mice did not change significantly. Differences in the production of these cytokines may affect the immune response to the vaccine in different strains of mice. From the results of TLR4 response pathway experiments, although the statistical analysis results showed that there was a significant difference between the IFN-β of ICR mice and the IL-6 of BALB/c mice before and after immunization, we found that this difference was not particularly obvious. Further repeated experiments can be carried out to verify this result. In another study, we conducted bacterial challenge experiments with three types of aP vaccine-immunized mice and found that the phenotypic differences in immune responses among the strains were consistent with the trends in TLR4 genotypic differences [29]. Additionally, this study revealed varying degrees of protectiveness against bacterial attack among the three mouse strains, with BALB/c mice exhibiting the poorest protection. This correlates with the upregulation of the IL-6 cytokine observed in this study’s BALB/c mice. In the immune response of this strain, the upregulation of IL-6 likely plays a more significant role as a chemoattractant and inflammatory factor. This is in accordance with the findings of Connelly et al. [30]. Interestingly, in studies related to human pertussis and immune responses [9], significant interactions among genes in the TLR pathway have been identified in inducing vaccine-induced immunity. These interactions highlight the functional relevance of these genes and their formation of genuine biological relationships within protein–protein interaction networks. In fact, many of the findings can be explained by genetic variations in proteins that directly or indirectly interact with antigen-presenting cells, B cells, or both, extracellularly and intracellularly. The fine-tuning of interacting proteins in the TLR pathway is crucial for inducing an optimal vaccine response. The production pathway of cytokines has not been fully studied. Therefore, excessive interpretation of cytokine results should be avoided in the analysis.

While our research provides important insights into TLR4 responses to PT in mouse models, we acknowledge certain limitations that may affect the broad applicability and interpretability of our results. Firstly, our study is primarily based on in vitro experiments conducted under controlled conditions, which may not fully mimic the complexity of the in vivo environment. Consequently, the in vitro responses we observed may not directly translate into immune responses following live vaccine administration. Moreover, our research focused on cytokine responses at specific time points, which may have limited our comprehensive understanding of the dynamic changes in immune responses. Secondly, the scope of our study’s data are relatively limited. We only examined three mouse strains with a small sample size, which may limit the statistical power of our findings and their extrapolation to other mouse strains or humans. Additionally, our study did not include long-term follow-up experiments to assess long-term immune responses and protective effects after vaccination.

In light of these limitations, we will include a broader range of mouse strains and larger sample sizes in future studies, as well as conduct long-term in vivo experiments to validate our in vitro findings. Furthermore, future research will further explore the direct link between TLR4 genotype and in vivo vaccine responses, as well as how different mouse strains affect vaccine immunogenicity and protective efficacy. Through these additional studies, we can gain a more comprehensive understanding of the role of TLR4 in vaccine responses and provide stronger scientific evidence for vaccine development and evaluation.

## 5. Conclusions

In this study, the TLR4 genotyping of different strains of mice was detected, and the difference in TLR4 expression level and TLR4 pathway-related cytokines in spleen lymphocytes stimulated by PT antigen was preliminarily explored. Our results indicated that rs13489092, rs13489093, and rs13489097 of TLR4 may affect the antibody level produced by the pertussis vaccine in mice. To prove this correlation, further data support is needed. Under the stimulation of PT antigen, the degree of TLR4 gene expression inhibition and the activated downstream pathways were different in different strains of mice. The identified SNPs of the TLR4 gene in different mouse strains might account for the differences in cytokines levels determined after PT stimulation. Our studies might provide useful referees to reduce the mouse-derived difference in the determination of vaccine titer and increase the vaccine comparability from different origins, as different mouse strains were used for vaccine development in different countries.

## Figures and Tables

**Table 1:** Primers used for TLR4 genotyping.

Nu	Primers 5′→3′	SNP Loci
TLR4 Region 1	5′-ATAGGTGGACAATATGTTGAGTGG-3′	rs13498470
	5′-GTAGAGGTTAGAATTGCCCTTAGAT-3′	
TLR4 Region 2	5′-CTTGATACTGACAGGAAACCCTA-3′	rs27883166 rs13489091 rs13489092 rs13498475 rs13489093
	5′-TCGCCAAGCAATGGAAC-3′	
TLR4 Region 3	5′-GATGAATACCTCCTTAGTGTTGG-3′	rs4224524 rs13489097 rs27883161 rs3023006
	5′-TCGGTCCATAGCAGAGCC-3′	

**Table 2:** TLR4 genotyping of three strains of mice.

SNP Loci	Gene	ICR	NIH	BALB/c	Protein Changes
rs13489092	TLR4	Homozygous without mutation	heterozygous mutation	Homozygous missense mutations	M(ATG)→I(ATA)
rs13489093		Homozygous without mutation	heterozygous mutation	Homozygous missense mutations	V(GTC)→I(ATC)
rs13489097		Homozygous without mutation	heterozygous mutation	Homozygous missense mutations	R(CGC)→H(CAC)

**Table 3:** Gene expression of TLR4 in different strains of mice before and after PT stimulation.

Mouse Strains	PT Stimulation	Medium
ICR	0.033 ± 0.018 ^a^	1.088 ± 0.166 ^a^
NIH	0.038 ± 0.014 ^a^	0.926 ± 0.246 ^a^
BALB/c	0.01 ± 0.005 ^a^	1.085 ± 0.288 ^a^

Note: ^a^ indicates that the expression level of the TLR4 gene in mouse spleen lymphocytes was significantly different before and after PT stimulation. Sample size: *n* = 10.

## Data Availability

Data are provided within the manuscript.
